# Sensor detection and pacing for impending reflex syncope

**DOI:** 10.1093/europace/euag070

**Published:** 2026-05-18

**Authors:** Richard Sutton

**Affiliations:** Department of Cardiology, National Heart and Lung Institute, Imperial College, London W12 0HS, UK

**Keywords:** Closed loop system/stimulation, Rate Drop Response, Reflex syncope, Cardiac pacing, Vasovagal syncope, Carotid sinus syndrome

## Abstract

**Aims:**

Reflex syncope occurs infrequently but may benefit from pacing intervention. However, reflex syncope poses special problems for successful cardiac pacing. Between episodes, monitoring by the implanted device is required to provide warning of an impending syncope and initiate pacing therapy if appropriate. This article aims to review available devices and their function in different forms of reflex syncope.

**Methods and results:**

The first device dedicated to address detection of VVS was the Rate Drop Response (RDR) (Medtronic Inc., MN, USA). It used a modified rate hysteresis approach to detect falls in heart rate that are faster than physiological but slower than those in cardiac conduction tissue disease. Since the introduction of RDR more has been learnt about the development of vasodepression and cardiac inhibition in vasovagal syncope (VVS). Detection of vasodepression, which occurs substantially earlier than cardioinhibition, assumes great importance. Currently, such detection is only offered by the Closed Loop System (CLS) (Biotronik GmbH, Berlin, Germany). Carotid sinus syndrome (CSS) shows different haemodynamic features from VVS with early vasodepression absent which demands rethinking of device selection and programming. RDR yielded improved management of both VVS and CSS. CLS has demonstrated clear benefits in two important recent randomized controlled VVS pacing trials. So far, no comparative trial between RDR and CLS has been undertaken.

**Conclusion:**

Pacing the bradycardia of reflex syncope requires a full appreciation of its haemodynamic events, including timing of vasodepression and cardioinhibition requiring sophisticated detection and therapy delivery.

## Introduction

Prior to the early 1990s, little attention was given to pacing any condition other than cardiac conduction tissue disease, atrioventricular block and sinus node disease. Nevertheless, Fitzpatrick *et al*.^1^ in London had already shown that standard ventricular pacing (VVI mode) was problematic in vasovagal syncope (VVS)^1^ as had Morley *et al*.^2^ in Carotid sinus syndrome (CSS) demanding consideration of dual chamber pacing. The advent of tilt-testing for diagnosis of unexplained syncope^3^ allowed appreciation of not only a more appropriate pacing approach^4^ but fundamentally provided the opportunity to observe VVS in the laboratory using beat-to-beat haemodynamic monitoring. These induced attacks were confirmed by the patients to reproduce their spontaneous episodes suggesting that what could be observed had veracity. Early reports of pacing in VVS using rate hysteresis and DDI mode were relatively promising.^5,6^

## Pathophysiology and development of detection algorithms

Inspection of the heart rate behaviour recorded in tilt-induced cardioinhibitory vasovagal episodes prompted the invention of a modified rate hysteresis pacemaker that would detect a fall in heart rate that was neither the precipitous fall characteristic of the onset of atrioventricular block or asystole (although pacing response for the latter conditions was retained) nor the slowing of normal sinus rhythm on relaxation or in sinus arrhythmia.^7^ Use of this device was well reported in VVS patients.^8^

RDR has been included in hundreds of thousands of pacemakers although surely only used in a much smaller proportion and was copied by other manufacturers than the patent holder Medtronic Inc., Minneapolis, MN, USA.

Concurrent development of a sensing system by Biotronik GmbH, Berlin, Germany, beginning also in early 1990s, using right ventricular impedance detected from subthreshold current injections during systole at the tip of the right ventricular pacing lead (unipolar).^9^ The general aim of this development, called Closed Loop System or Stimulation (CLS), was to detect need for an appropriate pacing rate at any particular time (rate responsive pacing). It was conceived as a detector of myocardial contractility. This development gained market share slowly at first.

Although the potential of CLS to detect impending VVS was not widely appreciated, the Cardiology group in Aarhus, Denmark began a trial to test the benefits in VVS patients with the support of Biotronik, Denmark. However, the group experienced difficulty in recruiting patients causing them to approach the United Kingdom for help with this. Unfortunately, this spread word to Biotronik’s head office in Berlin where the project was cancelled. The explanation for this cancellation was that Biotronik did not want its CLS device to become known as the ‘syncope pacemaker’.

CLS pacing had already been used in a substantial number of VVS patients with good results. A small single-blind trial was published with outstanding results generating some skepticism.^10^ Fifty patients were included, all with severe recurrent cardioinhibitory VVS but they were not randomly distributed with only 9 in DDI mode at 40 ppm and 41 with CLS mode. The larger number of patients with CLS mode had no syncope recurrence in follow-up of 19 months but recurrences were frequent in those with DDI back-up pacing.

The work of van Dijk and colleagues in Leiden, NL focused attention on the blood pressure (BP) fall preceding VVS.^11,12^ They showed that during a positive tilt-test BP fall, termed vasodepression, began up to eight minutes, before heart rate fall (cardioinhibition). Others have found similarly long times^13,14^ offering confirmation of the observation and emphasizing the importance of early vasodepression detection in VVS.

Typically, VVS occurs in the upright position, either standing or sitting. This body position induces the pooling of blood in the lower part of the body which appears to be predominantly in the splanchnic bed.^15,16^ Stewart et al have shown that the greatest fall in impedance is in the lower abdomen during a tilt test^15^ while Tajdini’s group has shown that efficient lower limb support stockings do not improve resistance to recurrence of VVS.^16^ After a prolonged period of progressive vasodepression, about 8 min, the vagal aspect of VVS is triggered and cardioinhibition supervenes accelerating the BP fall. Exactly what triggers this remains ill-understood. Some emphasize a fall in Epinephrine^17^ and others consider sympathetic withdrawal more important.^13^ Epinephrine behaviour in VVS has been well-documented^17^ and is attributed to a reaction to the reducing venous return but its exact timing of rise and fall is unknown as very frequent sampling is needed in a large number of patients (to overcome a range of individual variability). Such intensive sampling renders a study more demanding and expensive which is likely to be the reason that it has not been performed.

The findings of Palmisano *et al*.^14^ and Russo *et al*.^18^ during tilt studies of CLS pacing, indicate that a fall in BP is associated with onset of CLS pacing, probably slows the further BP fall and may prevent cardioinhibition resulting in a prolonged duration of the episode. However, syncope or near syncope was not always prevented which should not be surprising as a positive tilt reveals an innate tendency to VVS^19^ rather than testing the effect of therapy. Thus, persistence of tilt positivity is compatible with no syncope recurrence in everyday life and the success of therapy. Prolongation of symptomatic warning of an impending attack, by delaying BP fall, may prompt the patient to take evasive action, thus, avoiding syncope or injury.

**Figure 1:**
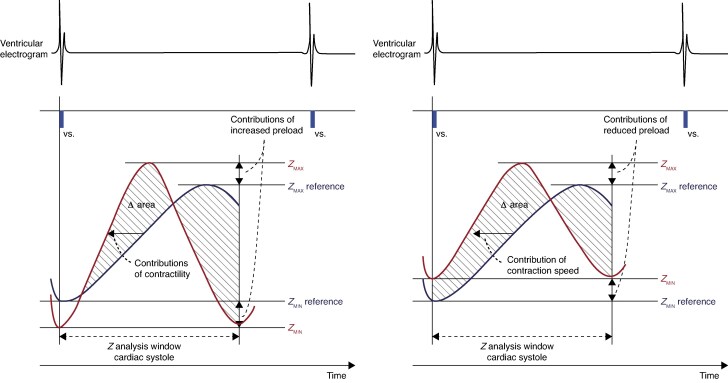
Mechanism of function of CLS.

The above discussion is relevant to detection of impending VVS by right ventricular impedance as employed by CLS. The early understanding of this function was that the increased contractility of the ventricles, prompted by rise in epinephrine, was the detected parameter. More recently, with the evidence of vasodepression beginning so long before cardioinhibition^11–13^ some rethinking has been undertaken by the manufacturer^20^ but without data on the timing of epinephrine rise and fall, a final answer cannot be given. If the onset of CLS pacing is triggered by a response to falling venous return with diminished RV volume, it is logical to consider this change is the primary trigger rather than contractility increase as a response to epinephrine release. Contractility detection is determined by examination of the rate of rise of pressure detected in the chamber. Explanations are given by Russo *et al*.^18^ but they are more considered by Gargaro *et al.*,^20^  *Figure 1*. Their figure legend, here reproduced with permission of Biotronik, explains the different functioning of the CLS algorithm in normal circumstances (left panel) and with reduced pre-load as is relevant to VVS (right panel).

Functioning of CLS under different stress conditions. The left panel is a schematic presentation of increased blood volume in late diastole (increased pre-load), which is expected to correspond to a relatively lower initial impedance value (Zmin on the red curve) compared with the reference trend (the blue curve). In this scenario, increased contractility according to the Frank-Starling principle causes the impedance to rise more rapidly and reach a maximum value (Zmax) earlier.

The right panel depicts reduced pre-load in late diastole due to decreased venous return, which is anticipated to correlate with a relatively higher initial impedance value (Zmin) compared with the reference trend. Compensatory mechanisms of cardiac output to counteract the drop in BP lead to an initial increase in heart rate, which enhances myocardial contraction speed through the force-frequency relationship and results in a faster and earlier rise in systolic impedance up to the maximum impedance (Zmax). Δ area = the area between the blue (current) and the red (reference) impedance curves. CLS, closed loop stimulation. Source Biotronik Gmbh, Berlin, Germany

**Figure 2:**
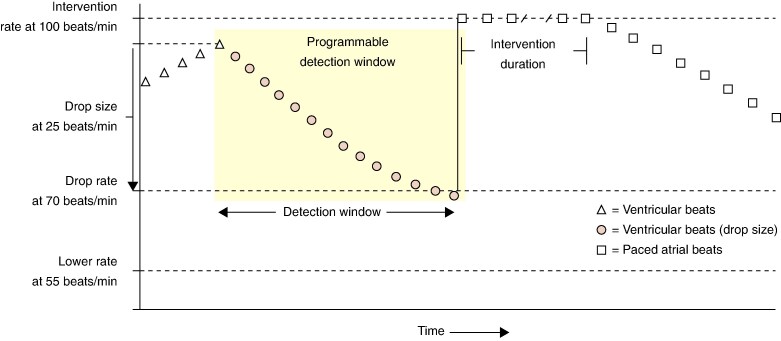
Mechanism of rate drop response.

CLS detection of impending VVS is also relevant to the RDR algorithm which hinges solely on heart rate fall. RDR has a programmable detection window which will trigger pacing if the rate of fall in heart rate is sufficiently fast to pass through the bottom of the window (window sill), *Figure 2*. Less steep falls in heart rate will pass through the side of the window when triggering of pacing is withheld. After passing through the window sill a programmable number of beats is required to confirm that the bradycardia persists. The effect of this design is that pacing can never start during vasodepression and that pacing, when triggered, will be further delayed by awaiting confirmation beats. Thus, RDR is quite compromised in treating VVS. For those patients with earliest onset of cardioinhibition, it can be expected to help and its programmable therapeutic pacing rate may also be valuable.

A detection window is shown highlighted in yellow. The window opens with the onset of rate fall. A Drop size of 25 bpm has been programmed. The falling rate passes the programmed Drop rate at 70 bpm and thus falls through the bottom of the window (window sill). Confirmation beats are not illustrated but one occurs below 70bpm triggering intervention pacing at 100 ppm which continues for a programmed duration and then gradually slows allowing sinus rhythm again to dominate. (Source: Medtronic Inc.)

The pacing rate in CLS’s response to an impending VVS is determined solely by the sensor. Although there are programmable upper and lower rates, the upper rate may not be reached in any triggered episode. It has been mooted that CLS should have something similar to the fast therapeutic rate of the RDR in the future.^18^

Another sensing system worthy of consideration is the Peak Endocardial Acceleration (PEA) of the Sorin Biomedica company which is now MicroPort. It was introduced in the late 1990s with encouraging initial results.^21,22^ However, little has been published on results in VVS detection in subsequent years. Both these studies of small numbers of patients showed that detection by PEA of VVS will occur about 2 min before collapse which may consistently be sufficient by introduction of pacing to alter or abort syncope but the time offered by the CLS algorithm allows even earlier onset of pacing.

## Carotid Sinus Syndrome

The pathophysiology of CSS differs from VVS.^23^ In the laboratory, it can be simulated by carotid sinus massage (CSM) although it is not known how well this reproduces spontaneous events. On CSM, cardioinhibition occurs within seconds of the onset of massage. If severe, intense bradycardia and asystole may be expected. The fall in heart rate is fast and equivalent to that seen on tilt in VVS at onset of cardioinhibition. It is accompanied by vasodepression which also may be severe but notably lasts a substantial time after CSM is complete in the prescribed 10 s.^24^

**Figure 3:**
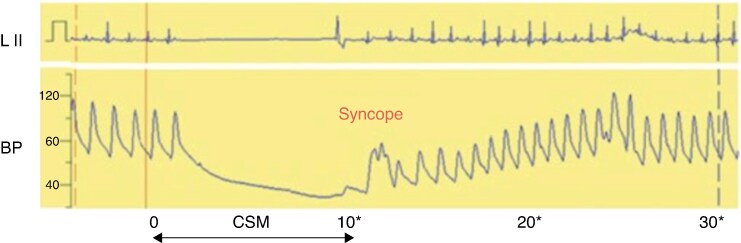
Example of carotid Sinus massage.

This typical example of CSM in a patient with CSS shows asystole beginning after 2 sinus beats from the onset of massage. At the conclusion of 10 s of massage, the heart restarts quickly but it takes a further 15 s for the BP to recover which demonstrates the vasodepression component of CSS. This can occur without significant bradycardia. *Figure 3* Reproduced with permission from the European Society of Cardiology.

Vasodepression in patients with severe cardioinhibition is estimated to occur in 20% when the generally accepted criterion of BP fall >50% is applied.^24,25^ However, Solari *et al*.^25^ found that the 50% criterion is inadequate to include all affected patients; they determined that a fall in systolic BP below 85 mmHg is a more appropriate vasodepression threshold in patients with or without severe cardioinhibition. Thereby, the great majority of those requiring therapeutic help with BP support or reduction in hypotensive medication is included. The timings of these haemodynamic events are variable. The duration of bradycardia is terminated by the cessation of CSM.

It is understood that rate hysteresis pacing is adequate in most CSS patients whose symptoms justify pacing.^2^ RDR can be anticipated to be effective for overcoming cardioinhibition and its post-event programmable higher rate of pacing may add value in combatting the persistence of vasodepression. However, RDR programming will be different from that needed in VVS, notably with a higher trigger rate (the window sill) and the absolute minimum of number of confirmation beats (or 1 only). CLS may also be effective in CSS although the reported experience has been small. In one case report of CSS complicating a neck tumour^26,27^ CLS appeared superior to simple DDD pacing. This finding begs the question of how CLS achieves this superiority. In CSS, there is no evidence of epinephrine release or increase in contractility but there is vasodepression which must reduce right ventricular filling and volume. Once again, one must suspect that the volume detection of CLS is the important factor as the contractility increase seen in VVS is absent.^26,27^

## Selection of patients for invasive therapy

It will be seen from the above that tilt as a method of investigating patients with syncope is invaluable as it permits reproduction of the reported events in the laboratory. Tilt was introduced in 1986^3^ and has stood the test of time even becoming more greatly endorsed in recent times.^28,29^ Notably, the attack must show cardioinhibition to justify pacing or cardioneuroablation. It may be argued that long-term ECG recording by implanted loop recorder is better because spontaneous episodes are shown but in the case of cardioinhibition there are no reported differences compared with tilt.

## Pacing in VVS

The focus here is on recent better designed randomized controlled trials (RCT) in VVS.

Pacing for VVS has largely seen RCT comparisons of DDD mode with RDR against a back-up mode such as DDI at 30 ppm or even ODO which is sensing only. Of these, the ISSUE 3 trial, the most recent and largest of the VVS pacing trials up to 2013, using RDR,^30^ showed a clear benefit for pacing (DDD + RDR vs. ODO) in reduction of syncope recurrence.

In CLS pacing, two RCTs published with solid trial designs in the last 9 years have shown clear benefit for CLS over either DDI 30 ppm or ODO.^31,32^ The double-blind SPAIN study^31^ included 46 patients older than 40 years with severe recurrent cardioinhibitory VVS (positive cardioinhibitory tilt tests) that were randomized to either 12 months of CLS mode or 12 months of DDI at 30 ppm. Four patients in CLS had syncope recurrence while 21 in DDI experienced these (*P* = 0.017). The time to first syncope recurrence was 29 months in CLS and 9 months in DDI (*P* < 0.0001). The BioSync double-blind trial^32^ of 128 patients older than 40 years, with severe recurrent cardioinhibitory VVS (again documented by tilt) showed 10 syncope recurrences at 11.9 months in CLS vs. 34 in ODO (*P* < 0.00005).

There has been no direct comparison between RDR and CLS although a protocol for such a trial has been published.^33^ It appears that neither Medtronic nor Biotronik is willing to fund such a trial rendering it only possible if funded by a neutral body such as the Medical Research Council in UK.

The latest guidelines from the European Society of Cardiology (ESC) in 2021^34^ determine pacing for VVS patients over 40 years of age with intractable symptoms and documented bradycardia (cardioinhibition) as a Grade I Level of Evidence A indication. These guidelines make no recommendation of choice of detection algorithm. The latest statement on pacing by a group of US professional committees addresses appropriate use criteria rather than claim to be guidelines.^35^ The document offers minimal mention of reflex syncope (Neurogenic syncope with a profound cardioinhibitory response is the term used) under leadless pacing stating that this may be appropriate.

## Pacing in CSS

In treatment of CSS by pacing, there have not been any randomized controlled trials of sufficient numbers to draw conclusions on the superiority of any mode of pacing assessed by Wieling and co-workers, published in 2011^36^ with no trial since that report. However, a large and long series of 164 patients was published in 2014^37^ showing quite good results only marred by syncope recurrence of 7% which was attributed to vasodepression over mean follow-up of 39 months. The mode of pacing was not stated. Further, a series of 158 patients all paced dual chamber with a longer follow-up of 89 months showed a syncope recurrence rate of 17% again attributed to vasodepression.^38^

ESC guidelines^34^ recommend dual chamber pacing for symptomatic cardioinhibitory CSS as a Grade I Level of evidence A. The recent US appropriate use document is silent regarding CSS.^36^

It is well known that CSS and VVS may coexist which was shown in the SUP studies.^39^ Using the approach recommended in those studies and reiterated in the recently published 2-Step method of investigation of syncope^39^ the second^29,40^ investigation is CSM after ambulatory BP monitoring. If positive, CSS is considered dominant which is logical in haemodynamic terms, as explained above. These patients are then paced without further study. For them, the choice of sensing system is as for CSS existing alone.

## Alternative treatments for vasovagal syncope

Cardioneuroablation (CNA) is expanding rapidly as a therapeutic option in VVS. It should be stated that a process should be undertaken prior to any invasive therapy.^41^ Now, this is particularly true for CNA as little controlled trial data is available in contrast to that in pacing for VVS. Once it is established that invasive therapy is the favoured option, consideration of the recent European Heart Rhythm Consensus on CNA should be given,^42^ plus the later registry report.^43^ This will be a quality approach to CNA which remains a therapy without long-term data. It must be added that the data for pacing only applies to patients of 40 years or more, established early in the evolution of pacing therapy because of a reticence to implant devices in younger patients. This impinges on practice of CNA which is currently more widely adopted in younger patients.

## Conclusions

This paper has reviewed the pathophysiological basis for detection of impending VVS, the development of algorithms to detect these changes and the available scientific evidence of their efficacy. CSS presents different haemodynamics where different programmes and even algorithms need consideration. There remain many important questions unanswered with little hope that the future will bring these answers.

It is reasonable to assume that no head-to-head trial between RDR and CLS will be mounted which leaves the choice between them to be based on the pathophysiology described above. Thus, the importance of studying pathophysiology and teaching its relevance should be an essential aspect of cardiology training allowing the physician to make an informed choice of sensor in pacing reflex syncope.

For the practicing clinician, today’s choice of sensor system is the CLS to obtain best results in managing VVS possibly also CSS by pacing.

## Data Availability

No original data is included; all data is available from the references.
